# High Prevalence of Intestinal Pathogens in Indigenous in Colombia

**DOI:** 10.3390/jcm9092786

**Published:** 2020-08-28

**Authors:** Simone Kann, Daniela Bruennert, Jessica Hansen, Gustavo Andrés Concha Mendoza, José José Crespo Gonzalez, Cielo Leonor Armenta Quintero, Miriam Hanke, Ralf Matthias Hagen, Joy Backhaus, Hagen Frickmann

**Affiliations:** 1 Department for Infectious Disease Diagnostics, Bernhard -Nocht Institute for Tropical Medicine, 20359 Hamburg, Germany; jessica_hansen@gmx.de; 2 Comprehensive Cancer Center Mainfranken, University Hospital of Wuerzburg, Translational Oncology, 97078 Wuerzburg, Germany; daniela.bruennert@gmail.com; 3 Institución Prestadora de Servicios de Salud Indígena, Dusakawi IPSI, Valledupar 200001, Colombia; gustavoconcha16@gmail.com (G.A.C.M.); josejosecrespog@gmail.com (J.J.C.G.); 4 Laboratorio Salud Pública Departamental, Valledupar 200001, Colombia; cielo_armenta@hotmail.com; 5 Institute for Medical Microbiology, Virology and Hygiene, University Medicine Rostock, 18051 Rostock, Germany; miriam.hanke@uni-rostock.de (M.H.); hf062@med.uni-rostock.de (H.F.); 6 Department of Microbiology and Hospital Hygiene, Bundeswehr Central Hospital Koblenz, 56072 Koblenz, Germany; RalfMatthiasHagen@bundeswehr.org; 7 Institute for Medical Teaching and Medical Educational Research, University Hospital Wuerzburg, 97070 Wuerzburg, Germany; Backhaus_J@ukw.de; 8 Department of Microbiology and Hospital Hygiene, Bundeswehr Hospital Hamburg, 20359 Hamburg, Germany

**Keywords:** protozoa infections, helminthes, bacterial infections, gastrointestinal infection

## Abstract

Background: Intestinal infections remain a major public health burden in developing countries. Due to social, ecological, environmental, and cultural conditions, Indigenous peoples in Colombia are at particularly high risk. Materials: 137 stool samples were analyzed by microscopy and real-time-Polymerase Chain Reaction (RT-PCR), targeting protozoan parasites (*Giardia intestinalis*, *Entamoeba histolytica*, *Cryptosporidium* spp., and *Cyclospora cayetanensis*), bacteria (*Campylobacter jejuni*, *Salmonella* spp., *Shigella* ssp./enteroinvasive *E. coli* (EIEC), *Yersinia* spp., enterohemorrhagic *E. coli* (EHEC), enteropathogenic *E. coli* (EPEC), enterotoxin-producing *E. coli* (ETEC), enteroaggregative *E. coli* (EAEC), and *Tropheryma whipplei*), and helminths (*Necator americanus*, *Strongyloides stercoralis*, *Ascaris lumbricoides*, *Ancylostoma* spp., *Trichuris. trichiura*, *Taenia* spp., *Hymenolepis nana*, *Enterobius vermicularis*, and *Schistosoma* spp.). Microscopy found additional cases of helminth infections. Results: At least one pathogen was detected in 93% of the samples. The overall results revealed protozoa in 79%, helminths in 69%, and bacteria in 41%. *G. intestinalis* (48%), Necator/hookworm (27%), and EAEC (68%) were the most common in each group. Noteworthy, *T. whipplei* was positive in 7% and *T. trichirua* in 23% of the samples. A significant association of one infection promoting the other was determined for *G. intestinalis* and *C. jejuni*, helminth infections, and EIEC. Conclusions: The results illustrate the high burden of gastrointestinal pathogens among Indigenous peoples compared to other developing countries. Countermeasures are urgently required.

## 1. Introduction

The indigenous tribe called Wiwa lives in retracted areas in the Sierra Nevada de Santa Marta in the northeast of Colombia. Since Wiwa communities avoid contact with the outside world, little is known, e.g., about their burden of gastrointestinal infections. According to the Wiwas, gastrointestinal complaints are the leading problem in their communities, causing high morbidity and mortality rates. The focus of this study was on bacterial and protozoan infections, as the area’s climate and poor socio-economic conditions (simple housing, lack of sanitation, etc.) provided favorable conditions for these pathogens. Furthermore, the Wiwa’s traditional mode of agriculture (top dressing), living together with livestock, and poor drinking water access (rivers and unprotected wells) likely contribute to high infection rates.

These kind of infections cause approximately 2 billion diarrhetic episodes worldwide and kill about 5.8 million children (2015) [1]. A further major analysis factor for this study was soil transmitted helminths (STHs), as they belong to the “Big Five” of neglected tropical diseases [2].

The main aim was to find evidence for and determine the prevalence and underlying causes of gastrointestinal infections in Wiwa communities for effective countermeasures to be implemented.

## 2. Material and Methods

### 2.1. Ethical Approval

The study was performed in line with the Declaration of Helsinki. It was approved by the Ethics Committee of Valledupar, Cesar, Colombia (Acta no 0022013). Written informed consent was obtained from each participant or the parent/legal guardian of a child prior to participation. All participants were informed about their results and received treatment, if appropriate.

### 2.2. General Information

In July and November 2014, 137 indigenous volunteers from the villages of Tezhumake (Department Cesar) and Seminke (Department La Guajira) provided stool samples. This corresponds to 80% and 50% of the respective populations living in these communities. Additionally, 13 samples came from Valledupar (Department Cesar), a larger city in the area. In total, 102 participants provided complete anthropometric and clinical data, and a physical examination was performed by a specialist for internal medicine. In 34 cases, individual pieces of information were missing (e.g., age or weight). These values were statistically treated as missing, which means that an imputation procedure was not conducted.

### 2.3. Nucleic Acid Extractions and Polymerase Chain Reactions (PCRs)

DNA was extracted using a QIAmp DNA stool mini kit (QIAGEN, Hilden, Germany), following the manufacturer’s protocol. Extracts were thereafter stored at −20 °C. A part of the stool samples was preserved in 5% formalin to perform the microscopic analysis. PCR investigations were carried out on site. In addition, extracts and formalin samples were sent to the Bernhard–Nocht Institute for Tropical Medicine (BNITM, Hamburg, HH, Germany). During the BNITM microscopy, control examinations and further analysis were performed. Airfreight requirements were fulfilled, the cooling chain was not interrupted (based on the temperature control documentation), and transport was done by a specialized company (World Courier, Frankfurt, HE, Germany).

In-house real-time multiplex PCRs for protozoan and helminthic parasites targeting *Entamoeba histolytica*, *Giardia intestinalis*, *Cryptosporidium* spp., and *Cyclospora cayetanensis*, as well as *Necator americanus*, *Strongyloides stercoralis*, *Ascaris lumbricoides*, *Ancylostoma* spp., *Trichuris trichiura*, *Schistosoma* spp., *Enterobius vermicularis*, *Taenia saginata*, *Taenia solium*, and *Hymenolepis nana* were performed as described before [3]. Further, enteroinvasive bacterial pathogens like *Campylobacter jejuni*, *Salmonella* spp., *Shigella* ssp./enteroinvasive *E. coli* (EIEC), and *Yersinia* spp. were assessed by in-house real-time PCR [4]. To identify diarrheagenic *E. coli* infections, Rida Gene RT-PCR assays for enterohemorrhagic *Escherichia coli* (EHEC), enteropathogenic *E. coli* (EPEC), enteroinvasive *E. coli* (EIEC), enterotoxin-producing *E. coli* (ETEC), enteroinvasive E. coli (EIEC), and enteroaggregative *E. coli* (EAEC) (R-Biopharm AG, Darmstadt, HE, Germany) were used, as described before [5]. Furthermore, a species-specific PCR for *Tropheryma whipplei* [6] was applied [7]. The DNA of phocid herpesvirus was included as an internal control for the in-house PCRs [8,9].

In all RT-PCRs runs, positive and negative controls were included: As positive controls, synthetically designed target sequences linked by EcoR1 endonuclease restriction sites and inserted into pEX-A128 vector backbones were used (Eurofins Scientific SE). Negative controls included PCT water samples that had undergone the whole nucleic acid extraction process to exclude sample contamination. All primers and probes of the in-house PCRs were purchased from Eurofins, Hamburg, HH, Germany. The assays were performed on a multi-channel RotorGene Q Cycler (Qiagen, Hilden, HE, Germany).

In addition, light microscopy of all stool samples by direct saline and/or iodine mounts and following a formol-ethyl acetate concentration was performed.

### 2.4. Demographic Data

To evaluate the average parameters related to size, weight, and growth, the official tables provided by the Organización Mundial de Salud 2006–2007 were used, which are valid for the Colombian population (most currently available tables). Individuals aged 0–19 years were scored by their weight–size, weight–age, and size–age tables, each corresponding to the male and female sex. The Body Mass Index (BMI) was calculated according to World Health Organization (WHO) recommendations.

### 2.5. Statistical Methods

Statistical analyses were carried out using R 3.6.1 (2020 Version 131073, Boston, MA, USA, Open Source) [10]. To compare the strength of association for various groups, the Mantel–Haenszel odds ratio (OR) with a 95% confidence interval (CI) was computed. To compare more than two groups, an Analysis of Variance (ANOVA) was calculated. The relation of the predictor and outcomes was analyzed using the logit model. For categorical data, Chi-Square test was employed. Z-scores for weight-for-age (wfa), length/height for age (hfa), and BMI-for-age based on the WHO Child Growth Standards were calculated using the R-package “zscorer”.

## 3. Results

### 3.1. Demographic Results

Of the 137 stool samples, 50% (68) were from female and 50% (69) from male volunteers. In the overall results, there was no sex dependency detectable (OR 1.24; 0.95-CI [0.36, 4.72]).

The average age was 24.6 years (SD = 18.16). Twenty-one samples were from children aged 0–6, 42 from adolescents aged 6–18, and 74 from adults older than 18 years. Stool consistency was normal in 18% (25) of cases; all other stool samples were fluid, pappy, or had a mucous texture. In 4 cases, the stool was additionally covered with blood (PCR showed in all cases an infection with *E. histolytica*).

The 137 questionnaires and physical examinations revealed that 88% (119) had no complaints, 10% (13) complained about diarrhea, 2% (2) noted abdominal pain, and 2% (2) suffered from diarrhea and abdominal pain. In one case, information was missing.

Of the 119 individuals who stated to have no complaints, only 7% (8) were negative in all tests. Of those who stated complaints, only 3% (4) were negative in all tests (which may be due to pathogens not included in the test panel). Whether persons stated complaints or not, only 9% (12) were negative in all tests performed. When using the reported complaints as a predictor, we found a sensitivity of 10% and a specificity of 60%. There was no statistical association between the subjective reporting of symptoms and having an intestinal pathogen X^2^ (1, *n* = 137) = 3.3, *p* > 0.05.

For individuals without complaints, 51% carried helminths, 39% bacteria and 55% protozoa. Of individuals with complaints, 59% carried helminths, 38% bacteria, and 60% protozoa.

**Figure 1:**
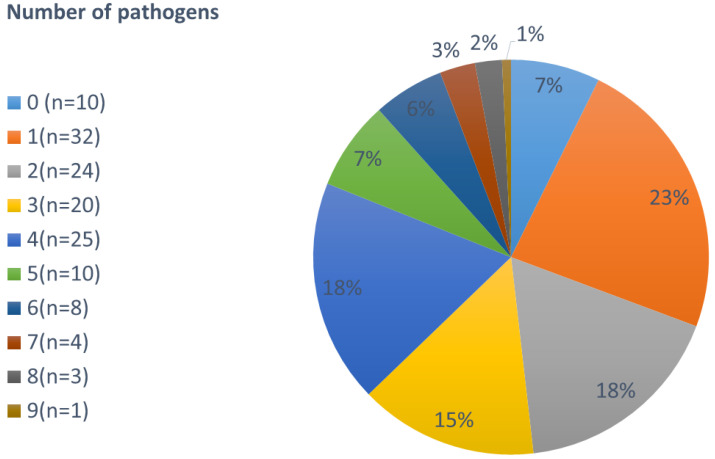
Number of pathogens found per person (not stratified by age).

The majority of volunteers (38%) complaining about diarrhea and/or abdominal pain carried 3–4 pathogens. Among these, the most frequent combination was *C. jejuni*, *G. intestinalis*, and EPEC accompanied by either *A. lumbricoides*, *T. trichirua,* or *E. vermicularis*. Taking the PCR and microscopy results together, at least one pathogen was found in 93% of the samples, at least two in 69%, three in 52%, four in 37%, five in 19%, six in 12%, seven in 6%, eight in 3%, and nine in 1% (see Figure 1).

The maximum of 9 pathogens (*C. jejuni*, *G. intestinalis*, *A. lumbricoides*, *T. trichiura*, *N. americanus*, *H. nana*, *E. vermicularis*, EPEC and EIEC) was found in a seven-year old boy.

**Figure 2:**
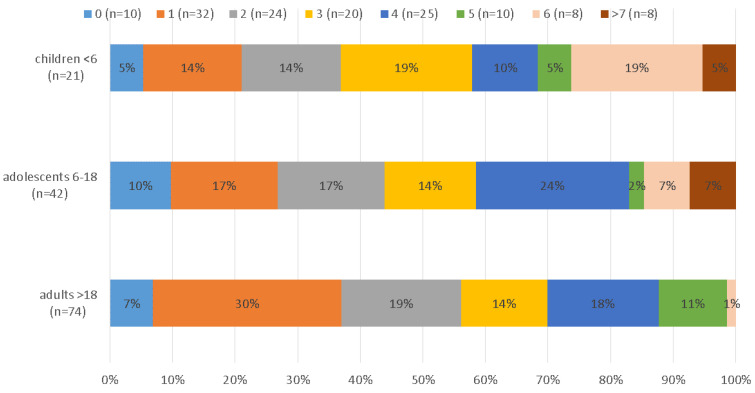
Number of infecting pathogens stratified by age in years.

Children had significantly more infections (M = 3.81, SD = 2.34) than adults (M = 2.5, SD = 1.65), with adolescents ranking in the middle (M = 3.19, SD = 2.28). Figure 2 shows the number of infections in each age group. An ANOVA could be computed since Levene’s test for the homogeneity of variance was not significant (*p* = 0.24).

**Figure 3:**
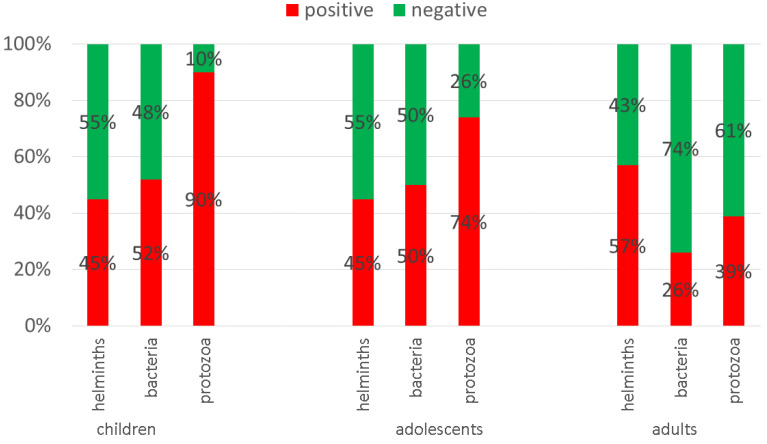
Type of parasite per age group (*n* = type of parasite based on at least one positive detection).

For protozoan parasites in particular, age was the most robust predictor: the older the individual, the less likely a positive result for protozoa. For more details, see Figure 3.

### 3.2. Co-Infections

Co-infections were common: Bacterial and protozoan pathogens were present in 31%, protozoan and helminth infections in 34%, and bacterial and helminth pathogens in 25% of the cases. Infections with at least one pathogen from all three classes were seen in 21%.

Applying logistic regression, we found that the presence of bacteria significantly predicted protozoa and vice versa. When analyzing the predictive power of single parasites, some notable observations of interest can be explored:

Co-infection with *C. jejuni* and *G. intestinalis* was common, as *C. jejuni* showed a predictive value of 67% for *G. intestinalis* infections and a value of 43% for *C. jejuni*. Further symbiotic interactions were found for *N. americanus*/hookworm and EIEC/*Shigella* spp., *A. lumbricoides,* and *T. trichiura*.

*S. stercoralis* symbiotically interacts with *T. trichiura* and *Necator*/hookworm pathogens. Evidence suggests that *S. stercoralis* infections tend to support EIEC infections, rather than vice versa.

Notably, EPEC supports EAEC (90%) and ETEC infections (56%), EAEC supports EPEC (55%) and ETEC (46%), while ETEC only supports EPEC (63%). Interestingly, this observation is not true for various other pathogens, such as *E. histolytica*, *E. nana*, and *Salmonella* spp. For more detailed information, see Table 1.

### 3.3. PCR Based Results

#### 3.3.1. Protozoa

Overall, the protozoan PCRs indicated 79% positive results. *G. intestinalis* was the most common pathogen (48%), followed by *C. cayetanensis* (12%). Seventeen of the positive results for *C. cayetanensis* occurred in children (59%); 50% of these were in those 1–4 years of age and 50% were in those 5–12 years of age. *E. histolytica* was found in 7% of the samples. *Cryptosporidium* spp. (1%) was only detected in one sample from a 37-year old man with an unknown immune status. He did not report any symptoms but was also positive for EPEC, *G. intestinalis*, and *C. jejuni*.

#### 3.3.2. Bacteria

The real-time-PCRs revealed positive results for bacteria in 41% of the samples. The leading bacteria within the *E. coli* group was EAEC (67%), followed by ETEC (53%), EPEC (42%), EHEC (20%), and *Shigella*/EIEC (18%). In the other bacterial panel, the most common pathogens were *C. jejuni*, *Salmonella* spp., and *T. whipplei,* with 31%, 5%, and 7%, respectively. *Yersinia* spp. did not occur.

#### 3.3.3. Helminths

As a leading helminthic infectious agent, *T. trichiura* was found in 23% of the cases, followed by *Hymenolepis nana* (21%), *N. americanus* (18%), *A. lumbricoides* (13%), Taenia (4%), *S. stercoralis* (4%), and *E. vermicularis* (2%). *Ancylostoma* spp. and *Schistosoma* spp. were not found.

### 3.4. Microscopy Based Results

The microscopy results showed *T. trichiura* (20%), hookworms (18%), *H. nana* (13%), *A. lumbricoides* (11%), *G. intestinalis* (3%), and *E. vermicularis* (2%). *N. americanus* was found to be positive in 25 PCR runs and 24 times via microscopy (classified as Hookworm, with 92% agreement, kappa 0.73, *p* < 0.001). For *A. lumbricoides,* the results were positive under PCR 17 times and 15 times under microscopy (with a 96% agreement, kappa 0.79, *p* < 0.001). For *G. intestinalis*, PCR revealed 66 positive results, while microscopy revealed only 4 (54% agreement, kappa 0.06, *p* < 0.05). Microscopy missed infections compared to PCR in 40% of the cases (false negative); positive results not detected in PCR occurred in 11% of the cases.

### 3.5. Villages

Stratified by village, there were 172 positive results in Tezhumake, 202 in Seminke, and 18 in Valledupar. In Seminke, 100% of individuals tested carried at least one pathogen, in Tezhumake 90%, and in Valledupar 85%. See Table 2 for further results. Of all pathogens found within the age group of children <6 years of age, 64% were bacteria, 21% were protozoa, and 15% were helminths. Of all bacteria found in children <6 years of age, *C. jejuni* was found in 13% and *Shigella* spp. 0%.

In total, 70 of the 137 volunteers (51%) showed a positive result in the PCR targeting helminths. Eighty-six-percent of the cases (37) from Seminke carried at least one helminth infection, 37% (30) from Tezhumake, and 23% (3) from Valledupar.

**Figure 4:**
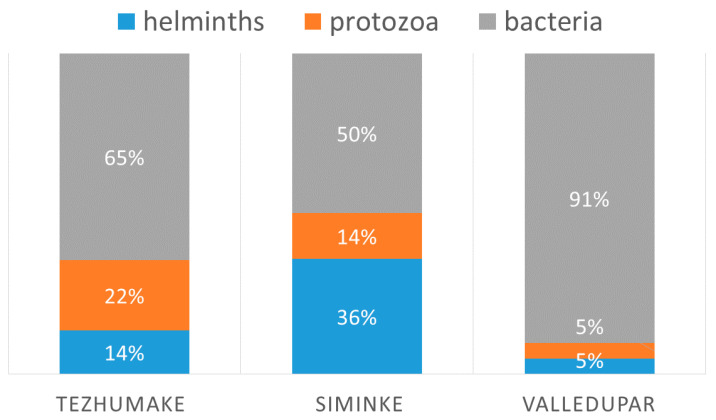
Comparison of infections related to villages.

Using logistic regression and referring to positive helminth cases as a subpopulation, we found that volunteers from Seminke were three times as likely to have an infection with helminths than volunteers from Tezhumake (X^2^ (2, *n* = 137) = 31.51, *p* > 0.001). Multiple infections with two or three pathogens were most common in Seminke (74%) (see also Table 2 and Figure 4).

### 3.6. Demographic Data

**Figure 5:**
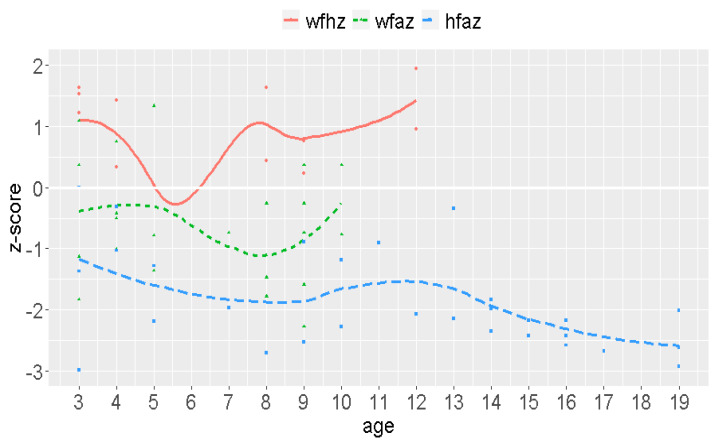
Weight-for-height (wfhz), weight-for-age (wfaz), height for age (hfaz) z-scores.

Weight ranged between 11.5 and 77.0 kg, with a mean weight of 43.6 kg (Md = 47.5; SD = 16.2). Weight stratified by sex revealed a mean weight of 42.4 kg for men and 40.8 kg for women. Height varied between 63 and 180 cm, with a mean size of 138.7 cm (Md = 145.0; SD = 24.5). Overall, 50% of the volunteers were ≤144 cm, and 75% were ≤ 153 cm. Height revealed a mean value of 132.6 cm for women and 139.36 cm for men (normal range for Colombians: 158.65 cm for women and 170.64 cm for man). The reference curves for height and weight revealed critical age stage results (see Figure 5) Low CT values in the PCRs, indicating a high pathogen burden, were found in most cases. The majority of children had a BMI above average for their age.

## 4. Discussion

The Wiwas live in very retracted areas of the Sierra Nevada de Santa Marta, north-east of Colombia. The burden of pathogens is much higher in this area than in most other developing countries. In total, 93% of the Wiwa population showed one or more infection(s). All 137 samples contained a total of 538 pathogens.

*G. intestinalis* is the most frequent protozoan parasite in developing countries [11,12,13,14]. In contrast to the average infection rates of 20–30% in Guatemala [15] and 13–43% in Haiti [16], the prevalence in our communities was 48%. Interestingly, only 50% of the infected individuals stated complaints, which may point to asymptomatic *G. intestinalis* carriers [17,18]. The higher prevalence found in younger children (81% of the infections) is in accordance with previous observations [19]. The observed differences in positive findings for *G. intestinalis* by microscopy and real-time PCR can be explained by the higher sensitivity of the well-established PCR technique [3] and is a generally well-known phenomenon [20]. Furthermore, the low sensitivity of microscopy might suggest pathogen concentrations close to or below the microscopic detection threshold, making low-replicative colonization more likely than high-replicative infection.

*E. histolytica* was found in 7% of the cases and was diagnosed by highly specific PCR, which is able to distinguish between *E. histolytica* and the pathogen forms *E. dispar/moshkovskii* [21,22]. Compared to studies performed in rural areas of Venezuela [23] and Mexico [24], where a prevalence of 11% and 14% was respectively detected, the occurrence rate among the Wiwas seems to be lower. The different diagnostic techniques applied (microscopy and different PCRs) might have affected these findings.

The rate of *C. cayetanensis* was 12% in the communities. In comparison, the prevalence of *C. cayetanensis* was 3–7% in Haiti and 2.3% in Guatemala [25]. In accordance with previous findings [26], the majority of infections were found in children and adolescents (42.8%). Notably, 20% were found in indigenous adults.

Overall, one protective predictor was found for protozoal infections: With increasing age, the infection rates decreased. A similar effect was shown previously for enteric colonization with *G. duodenalis* in asymptomatic Madagascan children [27].

Within the bacterial panel, the *E. coli* family was dominant. EAEC is described as the second most common agent (24%) [28]. It is an emerging pathogen affecting children and adults worldwide, causing acute and persistent diarrhea [29]. We found EAEC in 15% of the children and 58% of the adults.

ETEC causes infections in up to 200 million people worldwide, accounting for approximately 100.000 deaths per year [30]. While ETEC is the most common cause for travelers’ diarrhea, with rates in Latin America of 30%, 53% of our samples tested positive.

In Brazil, EPEC is considered to be the cause for 5–10% of all pediatric diarrhea cases [31]. In our study, it was present in 25% of the children and in 42% of the overall samples. EPEC can cause secretory diarrhea with mucus, severe water and electrolyte losses, and even bloody diarrhea. Hu et al. [31] noted that the frequency of EPEC infections decreases with age. The authors suggested the development of immunity or the loss of receptors with specific adhesins as the reason for this result. In contrast to this result, the number of infected individuals in our study was 65% of adults and 23% of adolescents. In addition to the typical EPEC (tEPEC), atypical EPEC (aEPEC) was found in diarrhetic patients of all ages [29]. The aEPEC or highly infectious doses required in adults (10^8^–10^10^ organisms) [29] could explain the high frequency of infections found in these age groups in our study. Furthermore, colonization needs to be considered [31].

*Shigella* spp. and EIEC have the same mechanism that causes diarrhea as they share virulence genes and are closely related [32]. In Brazil, the isolation of EIEC ranged from 0.5–15%, and in Bolivia, a prevalence of 2% was detected [29]. In our communities, the prevalence was 18.3%. As there is a high heterogenicity of EIEC isolates, the numbers of EIEC infections might be even higher than described [32]. Like many other pathogens, EIEC needs iron for bacterial colonization, so iron losses can be observed in infected patients [29].

EHEC/STEC belongs to a well-known group of foodborne pathogens distributed worldwide. Their ability to produce Shiga toxins makes them dangerous, and severe complications like Hemolytic Uremic Syndrome can occur [29]. While Gomes et al. [29] noted that the majority of patients are female (57%), with children below 5 years of age (54%) especially affected, we found children between 5–12 years of age (28% of the cases) to be most affected. Overall, we found positive results in 20% of the cases.

*C. jejuni* was found in 31% of the samples. *Campylobacter* infections are the main cause for foodborne bacterial illnesses and are even more common than *Salmonella*-associated foodborne diseases. Animals, especially chickens, as well as the environment, are promoting sources for such infections [33]. Chicken/poultry are the most common animals distributed within Wiwa communities. The consumption of raw milk might also contribute to the high number of infections [34].

In accordance with Young et al. [33], the prevalence for *Salmonella* spp. infections was lower than that for *C. jejuni* infections (5% and 31%).

*T. whippelei* was found in 7%. Since clinical manifestation could not be detected, colonization was assumed. In comparison to other developing countries, this rate is lower. For example, in Ghana, *T. whipplei* was found in 28% of children aged 2 months to 15 years [35]. Our results support the hypothesis of Vinnemeier et al. [35] that mainly children are colonized during the first years of life.

Thus far, helminth infections were described to be prevalent in Colombia, with rates of 0–9.9% [36]. In Wiwa communities, we found an overall prevalence of 69%. According to Pullan [37], about 1.5 billion people are infected with at least one soil-transmitted helminth [38], comprising 24% of the world´s population [39]. Therefore, STHs belong to the “Big Five” of neglected tropical diseases. The three leading STHs are *A. lumbricoides,* with 807–1.120 million infections per year; *T. trichiura,* with 604–795 million; and hookworm infections (*A. duodenale* and *N. americanus*), with 576–740 million [40]. These three were also the leading agents in our study, albeit in altered order (*T. trichiura*, hookworms, *A. lumbricoides*). The observations made by Moser [38] that *A. lumbricoides* tends to affect children under 12 years of age more frequently (20%) than older children (14%) and that *T. trichiura* infections are most common in patients older than 12 years (26%) compared to younger children (14%) could not be confirmed in our results. Infections were distributed equally across all age groups (see Table 2).

*Ascaris* and hookworm eggs mature in soil. Together with other worms (e.g., whipworms), the fecal–oral route is the most relevant one.

In contrast, hookworms release larvae that penetrate the skin, so the main route of transmission is walking barefoot on contaminated soil [40]. The threadworm is the most neglected tropical disease, although it is an exception among helminths, as it can cause endogenous autoinfections that lead to long lasting infections and systemic features with worm dissemination into various organs [41]. Brazil and Thailand are known to be hotspots for *Strongyloides* infections; the prevalence in Brazil is about 10–17%, while in Thailand, the overall prevalence is 24% [41]. We found 4% positive results, but these results did not come from multi-center studies using, e.g., Baerman’s method but instead from samples taken once on one day and analyzed with PCR only. In Venezuela, infection rates of 48% were reported in hospitalized patients, while only 3% rates were reported in community-based surveillance [41]. We found 4% positive results, which is high compared to 2%.

*H. nana* was found in 21% of the Wiwas. In a study performed in the rural zone of Colombia with children (5–15 years), 12% were infected [42]. Similar to our study, all age groups were affected.

The reasons for this high number of infections include bad water quality, low hygiene standards, and a lack of education. The consequences include gastrointestinal infections leading to permanent diarrhea, water and iron loss, electrolyte imbalances, anemia, protein deficits, malnutrition, stunted growth, intellectual impairment, cognitive and educational deficits, and complications in pregnancy (abortion, etc.) as documented (side) effects [2]. The crucial impact can be demonstrated by weight to age, weight to size, and size to age measurements. Children are in the percentile for age at −2/−3 below the average of 0, which is alarming. The average size of Wiwa women is 132.6 cm and 139.4 cm for men, while the normal sizes for Colombians are 158.7 cm for women and 170.6 cm for men. Although permanent and multiple gastrointestinal infections might be an underlying cause, other factors need to be considered, such as ethnic reasons.

The environmental impact becomes obvious when comparing the villages. While in Seminke 100% of the volunteers had at least one positive result, 89% had positive results in Tezhumake and 83% in Valledupar. Helminth infections were three times more common in Seminke than in the other villages. Although all communities have difficult living conditions, Seminke is the most distant from the next city. Furthermore, Seminke is surrounded by forest and Tezhumake by open grass-fields/steppes. Subsequently, the soil in Seminke stays moist longer making helminth transmission more likely.

The high number of co-infections is important to note. A significant association of one infection promoting the other was evident for *G. intestinalis* and *C. jejuni* (OR 3.00; 0.95-CI [1.40, 6.43]). Symbiotic interactions were determined for *Necator*/hookworm and EIEC/*Shigella* spp., *A. lumbricoides,* and *T. trichiura*. *S. stercoralis* seems to interact with *T. trichiura* and *Necator*/hookworm. Although the aforementioned interactions are symbiotic, this is not true for *S. stercoralis* and *EIEC* (see Table 2). Here, *S. stercoralis* supports EIEC unidirectionally. There are many potential reasons for the significant associations between infections. For example, *G. intestinalis* inducing dysbiosis in the intestinal microbiota might lead to an *G. intestinalis* expansion itself but might also produce favorable effects for other pathogens. In addition, immune-modulatory effects might contribute to co-infection interactions [43,44]. 

However, these findings only give tentative evidence due to the unequal sample sizes. Through the application of a propensity-score-approach for the identification of statistical twins [45], the results could be verified. Furthermore, logistic regression depends on varying sample sizes [46]. Subsequently, these findings need to be verified in larger scale studies.

Asymptomatic courses of intestinal infections are common in endemic regions [47], and many diseases have a broad range of clinical symptoms [48], but for such multiple and severe infections, it is difficult to believe that subjects do not experience complaints. Cultural socialization, however, supports the neglect of diseases like diarrhea. As most samples had a fluid or pappy texture, infected individuals might think that this is the normal consistency of stool. Furthermore, access to medical support is far away, with about a 6–12 h walking distance. If we consider (subjective) complaints to be a diagnostic test, the sensitivity would be 10% and the specificity 60%. However, when individuals stated complaints, 3–4 infections were present, with protozoal and/or helminth infections being the leading causes (60% and 58%).

In general, children were more infected than adults; in children, 2–3 infections most commonly occurred. Further, 90% of children below 6 years of age, 74% of the 6–18 year old volunteers, and 39% of the adults (above 18 years of age) suffered from protozoal pathogens. Among the co-infections, combinations of protozoa and helminths were the leading causes (34%), followed by protozoan and bacterial infections (31%) and bacterial and helminth pathogen combinations (25%). Infections with pathogens from all three groups were found in 21% of the cases.

Focusing on the limitations of the study, a further evaluation and classification of pathogens (stool culture, sequencing, etc.), as well as an analysis of water and soil, would be favorable. Due to financial limitations, these additional examinations could not be executed. Since not all inhabitants of the villages provided a stool sample, a selection bias cannot be excluded. However, due to the high compliance of volunteers (80% in Tezhumake and 50% in Seminke) and the results of the anamnesis and examination (feeling healthy and having no complaints), it is likely that no severe sampling error occurred. We also have to state that RT-PCR is not the optimum diagnostic procedure for the detection of *E. vermicularis*. The low proportion of detected *E. vermicularis* might be, in part, a consequence of insufficient sensitivity (56%) [3]. Thus, it is likely that infestations with low worm burdens may have gone undetected. From a technical point of view, the superiority of PCR compared to microscopy was confirmed, as microscopy missed infections (compared to PCR) in 40% of the samples [21,22,49,50,51].

The detected prevalence rates are alarming. The interplay of all the above factors accounts for the high burden of gastrointestinal diseases, confirming the observations made by the Wiwas themselves. The negative impact on indigenous communities is enormous. Death due to diarrhea is common, and other related diseases occur frequently (anemia, abortion, etc.). Counteractive interventions, such as drawing attention to public health and education, repetitive deworming programs, improvement of water quality, sanitation supply provisions, and permanent healthcare access are urgently needed.

## Figures and Tables

**Table 1:** Results of the logistic regression for single parasites. Significance: 0 ***, 0.001 **, 0.01 *. *C. jejuni* = *Campylobacter jejuni*, *EAEC* = *enteroaggregative E. coli*, *ETEC* = *enterotoxin-producing E. coli*, *EHEC* = *enterohemorrhagic E. Coli*, *EPEC* = *enteropathogenic E. coli*, *EIEC*/*Shig.* = *Enteroinvasive E. coli*/*Shigella* spp., *Giardia* = *G. intestinalis*, *E. histo* = *Entamoeba histolytica*, *Necator* = *N. americanus*, *S. stercoralis* = *Strongyloides stercoralis*, *Ascaris* = *A. lumbricoides*, *Trichuris* = *T. trichiura*, *H. nana* = *Hymenolepis nana*, *Enterobius* = *E. vermicularis*.

		Predictor Variables																
	Outcome variables (%)	1	2	3	4	5	6	7	8	9	10	11	12	13	14	15	16	17
BACTERIA	1. *C. jejuni* (*n* = 42)	1	0	24	31	17	25	45	42 **	56	29	41	60	43	47 *	55 **	0	33
	2. *Salmonella* spp. (*n* = 7)	0	1	7	12 *	9	9	0	71	11	12	3	0	10	0	7	0	0
	3. EAEC (*n* = 93)	69	86	1	84 **	87 *	90 ***	84	64	89	53	79	80	71	63	59	67	67
	4. ETEC (*n* = 53)	29	86 *	46 **	1	52	56 ***	48	44	44	47	38	40	33	34	41	67	67
	5. EHEC (*n* = 23)	1	29	19	22 *	1	57	10 *	15	11	6	12	0	19	13	10	33	17
	6. EPEC (*n* = 57)	2	71	80	55 ***	23	1	42	33	44	24	47	7	80	41	31	33	67
	7. EIEC/*Shig.* (*n* = 25)	1	14	52	23	16	17.4 *	1	17	22	18	47 ***	80 **	52	31 *	24	67	67
PROTOZOA	8. *Giardia* (*n* = 66)	67 **	8	38 *	45	44	39 *	42	1	44	53	41 *	40	38 *	44	76 ***	33	50
	9. *E. histo* (*n* = 9)	12	14	5	9	5	7	7	6	1	0	9	20	5	6	21 ***	0	0
	10. *Cyclospora* spp. (*n* = 17)	12	29	29 *	10	4	7	19	14	0	1	12	0	29 *	22	17	0	0
HELMINTHS	11. *Necator*/hookworm (*n* = 37)	33	14	67 ***	29	17	28	64 ***	21 *	33	24	1	80 *	67 ***	41 ***	17	33	50 *
	12. *S. stercoralis* (*n* = 5)	7	0	20	4	0	7	13 **	3	11	0	12 *	1	20	6	3	0	17
	13. *Ascaris* (*n* = 21)	21	29	1	16	9	19	52	12	11	35 **	41 ***	4.8	1	41 ***	14	33	10
	14. *Trichuris* (*n* = 32)	36 **	0	62 ***	22	17	23	40 *	21.2	22	41	52 ***	40 *	62 ***	1	38 *	67	33
	15. *H. nana* (*n* = 29)	38 **	29	19	18	13	16	28	33 **	67 ***	29	20	20	19	34 *	1	33	50
	16. *Enterobius* (*n* = 3)	0	0	5	2	4	2	8 *	2	0	6	4	0	5	6	3	1	17 *
	17. Taenia (*n* = 6)	5	0	10	4	4	7	16 **	5	0	0	12 *	20	10	6	10	33 *	1

Note. Logistic regression (logit model) allows one to analyze whether one variable predicts the other. Subsequently, three combinations are of interest: (1) Parasites, that determined each other to different strengths; (2) Parasites significantly predictive of various other parasites (e.g., Hookworm and Ascaris); and (3) Parasites, who do not predict other parasites at all (e.g., EHEC and EPEC to a certain extent).

**Table 2:** Gastrointestinal organisms identified in stool samples by species groups stratified by village and age groups. *C. jejuni* = *Campylobacer jejuni*, EIEC = enteroinvasive *E. coli*, EHEC = enterohemorrhagic *E. coli*, ETEC = enterotoxin-producing *E. coli*, EAEC = enteroaggregative *E. coli*, EPEC = enteropathogenic *E. coli*, *T. whipplei* = *Tropheryma whipplei*, *G. intestinalis* = *Giardia intestinalis*, *E. histolytica* = *Entamoeba histolytica*, *C. cayetanensis* = *Cyclospora cayetanensis*, *N. americanus* = *Necator americanus*, *S. stercoralis* = *Strongyloides stercoralis*, *T. trichiura* = *Trichuris trichirua*, *H. nana* = *Hymenolepis nana*, *E. vermicularis* = *Enterobius vermicularis*.

Organism	Village			Age Group in Years			Overall (*n* = 137)
	Semink (*n* = 43)	Tezhumake (*n* = 81)	Valledupar (*n* = 13)	<6 (*n* = 21)	6–18 (*n* = 42)	>18 (*n* = 72)	
Bacteria	49.8%	64.5%	90.5%	64.3%	64.4%	75.5%	60.2%
*C. jejuni*	16.5%	11.1%	50%	12.5%	13.6%	6.4%	17.6%
*Salmonella* spp.	1.7%	2.9%	0%	1.4%	1.5%	1.7%	2.2%
*Shigella* spp./EIEC	12.2%	6.4%	0%	9.7%	5.3%	4.7%	7.7%
EHEC	4.3%	8.2%	10.5%	5.6%	4.5%	5.6%	7.1%
ETEC	16.5%	18.1%	2.6%	9.7%	13.6%	11.1%	15.7%
EAEC	27.8%	31.6%	18.4%	19.4%	18.9%	23.1%	28.7%
EPEC	19.1%	17.5%	13.2%	9.7%	9.8%	15.8%	17.6%
*T. whipplei*	0.9%	2.9%	5.3%	29.2%	31.8%	31.6%	2.5%
Protozoa	14.3%	21.9%	4.8%	20.5%	17.1%	11.3%	17.3%
*G. intestinalis*	60.6%	75.9%	100%	73.9%	82.9%	57.1%	71%
*E. histolytica*	6.1%	12.1%	0%	4.3%	2.9%	20%	9.7%
*C. cayetanenis*	30.3%	12.1%	0%	21.7%	14.3%	20%	18.3%
*Cryptosporidium* spp.	3%	0%	0%	0%	0%	2.9%	1.1%
Helminths	35.9%	13.6%	4.8%	15.2%	18.5%	13.2%	22.5%
*N. americanus*	21.7%	19.4%	0%	23.5%	15.8%	36.6%	20.7%
*A. lumbricoides*	24.1%	2.8%	0%	23.5%	18.4%	24.4%	17.4%
*S. stercoralis*	4.8%	0%	50%	5.9%	5.3%	4.9%	4.1%
*T. trichiura*	28.9%	19.4%	50%	17.6%	39.5%	34.1%	26.4%
*H. nana*	12%	52.8%	0%	35.3%	31.6%	26.8%	24%
*E. vermicularis*	2.4%	2.8%	0%	5.9%	2.6%	2.4%	2.5%
*Taenia* spp.	6%	2.8%	0%	11.8%	2.6%	7.3%	5%
Total	42.9%	49.3%	7.8%	17.9%	32.7%	49.4%	100%
